# Vasoactive agents in acute mesenteric ischaemia in critical care.  A systematic review

**DOI:** 10.12688/f1000research.52782.1

**Published:** 2021-06-08

**Authors:** Christopher A Brennan, Peter Osei-Bonsu, Rachael Eimear McClenaghan, Ahmed Nassar, Patrice Forget, Callum Kaye, George Ramsay

**Affiliations:** 1 NHS Grampian, Aberdeen, UK; 2 University of Aberdeen, Aberdeen, UK; 3 University of Edinburgh, Edinburgh, UK; 4 Institute of Applied Health Sciences, University of Aberdeen, Aberdeen, UK; 5 Health Services Research Unit, University of Aberdeen, Aberdeen, UK

**Keywords:** Acute Mesenteric Ischaemia, AMI, Critical Care, General Surgery, vasoactives, vasopressors, infarction, ischaemic, perioperative care, splanchnic blood flow, noradrenaline, levosimendan, dobutamine, dopexamine, dopamine, milronone, systematic review, DELPHI, OVID, MEDLINE, EMBASE, COCHRANE, CDSR, Aberdeen, Grampian, Human

## Abstract

**Background**: Acute mesenteric ischaemia (AMI) is a surgical emergency which has an associated high mortality.  The mainstay of active treatment includes early surgical intervention, with resection of non-viable bowel, and revascularisation of the ischaemic bowel where possible. Due to the physiological insult of AMI however, perioperative care often involves critical care and the use of vasoactive agents to optimise end organ perfusion. A number of these vasoactive agents are currently available with varied mechanism of action and effects on splanchnic blood flow. However, specific guidance on which is the optimal vasoactive drug to use in these settings is limited. This systematic review aimed to evaluate the current evidence comparing vasoactive drugs in AMI.

**Methods**: A systematic search of Ovid Medline, Ovid Embase, Cochrane CENTRAL and the Cochrane Database of Systematic Review was performed on the 5th of November 2020 to identify randomised clinical trials comparing different vasoactive agents in AMI on outcomes including mortality. The search was performed through the Royal College of Surgeons of England (RCSEng) search support library. Results were analysed using the Rayyan platform, and independently screened by four investigators.

**Results**: 614 distinct papers were identified. After screening, there were no randomised clinical trials meeting the inclusion criteria.

**Conclusions**: This review identifies a gap in literature, and therefore recommends an investigation into current practice and clinician preference in relation to vasoactive agents in AMI. Multicentre randomised controlled trials comparing these medications on clinical outcomes will therefore be required to address this question.

## Introduction

Acute mesenteric ischaemia (AMI) is a time- critical surgical emergency, ^1^ where early diagnosis and management can prevent bowel infarction, multiorgan failure and death. ^2^^–^^4^ It is defined as a sudden inadequacy of arterial supply or venous drainage to the bowel, leading to ischemia and cellular damage, with or without necrosis. ^5^^,^^6^ AMI has an estimated incidence of ~1:1000 hospital admissions. ^1^^,^^4^^,^^7^^–^^9^

A number of pathophysiological mechanisms can lead to mesenteric ischaemia. ^4^ “Occlusive” mesenteric ischaemia is due to arterial or venous thrombosis or embolism. “Non-occlusive” is due to acute circulatory failure, usually in the critically unwell patient. ^10^^,^^11^ Non-occlusive mesenteric ischaemia (NOMI) can also occur in the setting of critical illness secondary to the use of vasoactive drugs because of splanchnic vasoconstriction. ^5^ Each of these processes cause a gut-derived systemic inflammatory response syndrome (SIRS) or mesenteric ischaemic necrosis, leading to severe metabolic derangement and culminating in multiple organ dysfunction requiring critical care intervention. ^12^^,^^13^

Early imaging with computerised tomography (with arterial and portal venous phase) ^6^ is important for diagnosis and instigating a timely management plan. The optimal management of AMI depends on the underlying pathophysiology and whether the affected bowel is ischaemic or infarcted. Treatment of AMI focuses on reperfusion and/or resection of non-viable bowel. ^14^ As in any critically unwell patient, adequate resuscitation of haemodynamic parameters is important to optimise end-organ perfusion and prevent the development of multiorgan failure.

Given the extent of sepsis response, AMI management usually requires critical care support. Vasoactive drugs are often required in this setting to optimise haemodynamic status, with the aim of improving supply to the end organs as well as optimising the perfusion of blood to the adjacent intestine segments to the area of ischaemia. ^15^ However, the choice of vasoactive agents is unclear for patients with AMI. This is a result of the various mechanism of action of these medications and differing levels of associated splanchnic vasoconstriction. Some agents, such as noradrenaline and adrenaline, can be effective in improving systemic vascular resistance and thus, maintain the perfusion pressure to the brain and heart. However, they can also be associated with profound splanchnic vasoconstriction which could exacerbate bowel ischaemia by precipitating NOMI. ^1^^,^^16^ Other drugs are perceived to have less of an effect on splanchnic vasculature and could theoretically improve perfusion to the primary area of pathology but may impact on perfusion pressure for other organs.

**Figure 1.**
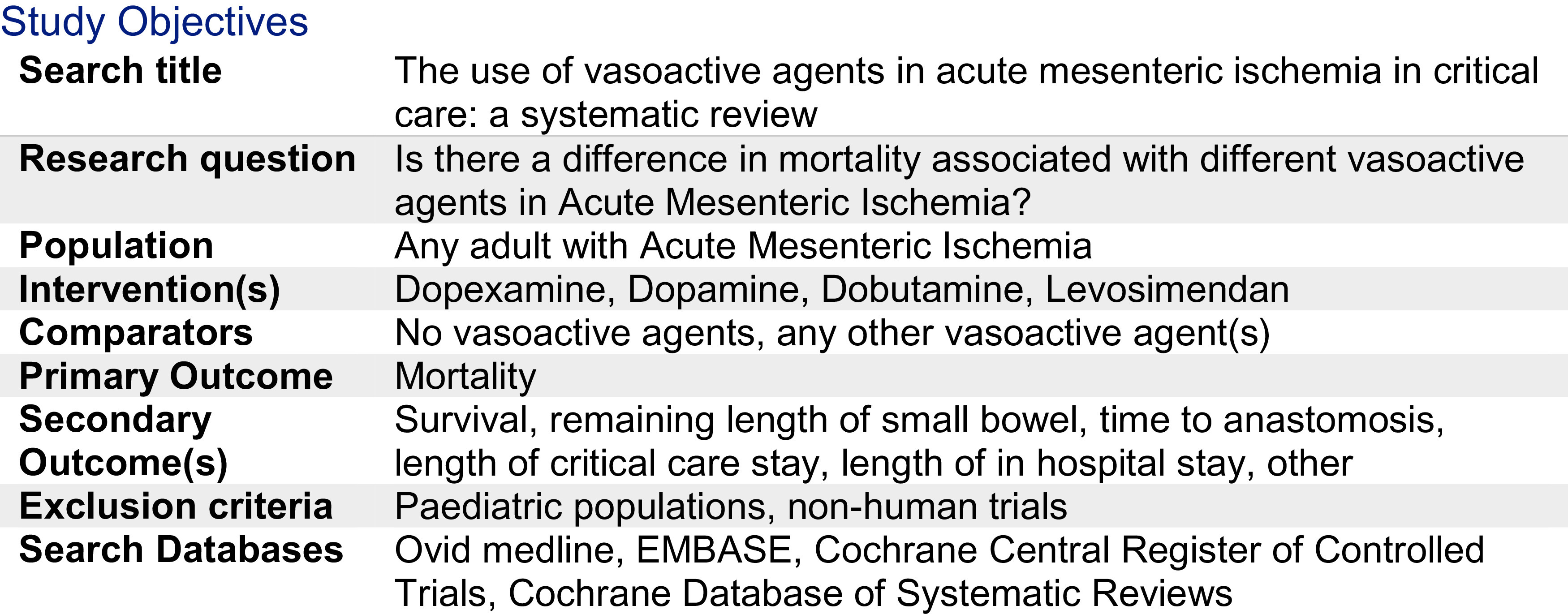
Summary of study objectives.

The mortality rate is variable but often high, especially when detected late or accompanied by metabolic derangement. ^4^^,^^7^ This variability in mortality may be secondary to differences in local practice ^5^^,^^17^^,^^18^ and between clinicians. It may also reflect the lack of evidence-based guidelines available for these conditions. ^19^^–^^23^ Vasoactive agents vary in their mechanisms of action, and balance of vasoconstriction, inotropy, and splanchnic vascular dilatation. It is not known whether one may be more beneficial than others in the setting of AMI. This primary aim of this systematic review is to evaluate the current evidence comparing mortality outcomes for vasoactive drugs in AMI. Our PROSPERO summary is illustrated in Figure 1.

## Methods

### Protocol and registration

This review has been prepared in line with the Preferred Reporting Items for Systematic reviews and Meta-analyses (PRISMA) statement. The protocol for this systematic review was developed and registered to PROSPERO prior to the analysis of search results ( CRD42020212291, 11/11/2020).

### Eligibility criteria

This systematic review aims to identify randomised clinical trials (RCTs) comparing mortality rates associated with different vasoactive agents in AMI. The target population were any patients with AMI admitted into a critical care environment. Vasoactive drugs included were noradrenaline, adrenaline, dopexamine, dobutamine, dopamine, levosimendan, vasopressin, ephedrine or phenylephrine. Comparators were either no vasoactive drug or any other vasoactive drug. The primary outcome was mortality. Secondary outcomes were survival, length of preserved bowel, time to anastomosis, length of critical care admission, and overall length of hospital stay. All published works were searched regardless of date of publication or language.

### Information sources

Searched sources were Ovid Medline, EMBASE, Cochrane CENTRAL and the Cochrane Database of Systematic Review.

### Search strategy

**Figure 2.**
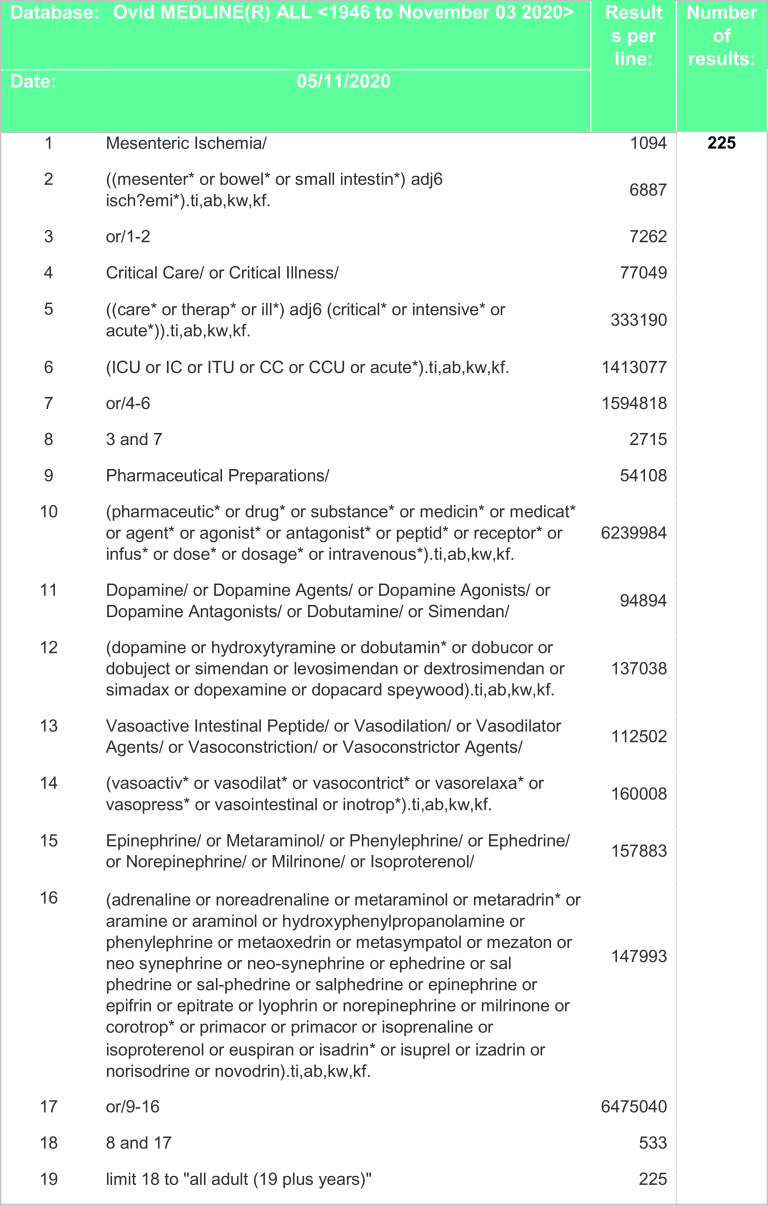
Search strategy – Medline.

**Figure 3.**
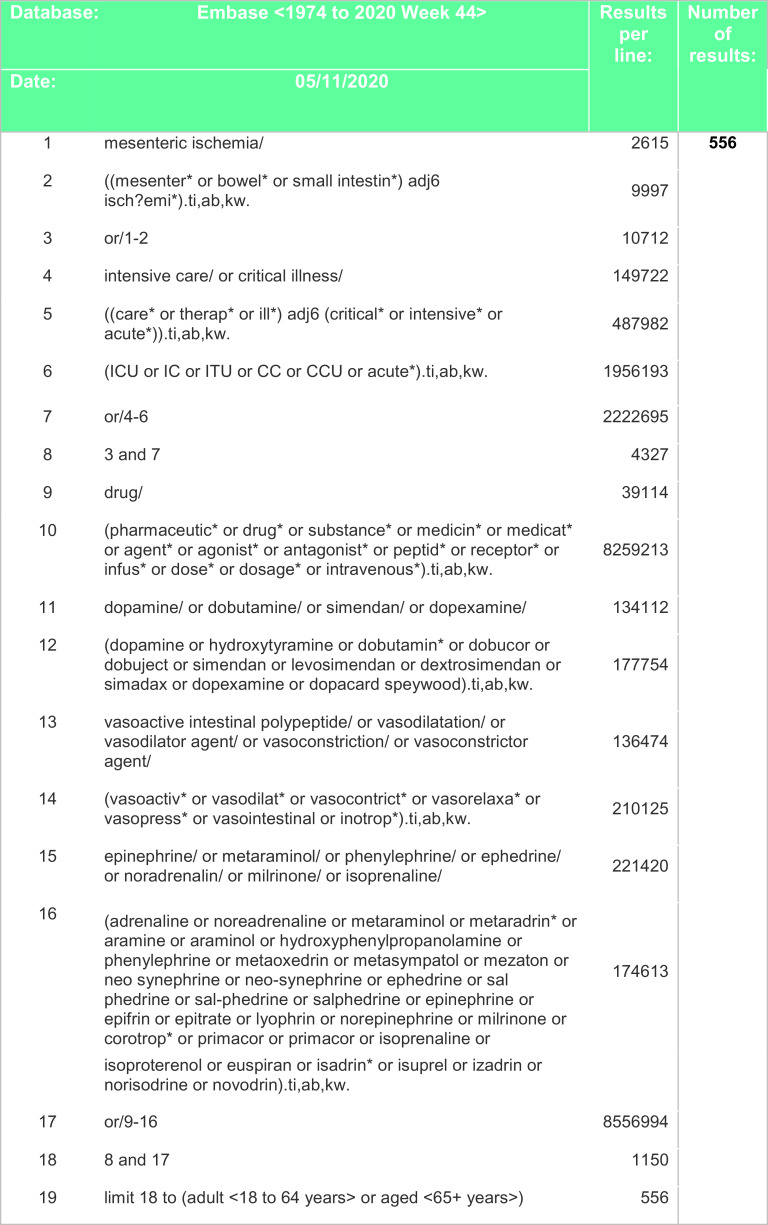
Search strategy – EMBASE.

**Figure 4.**
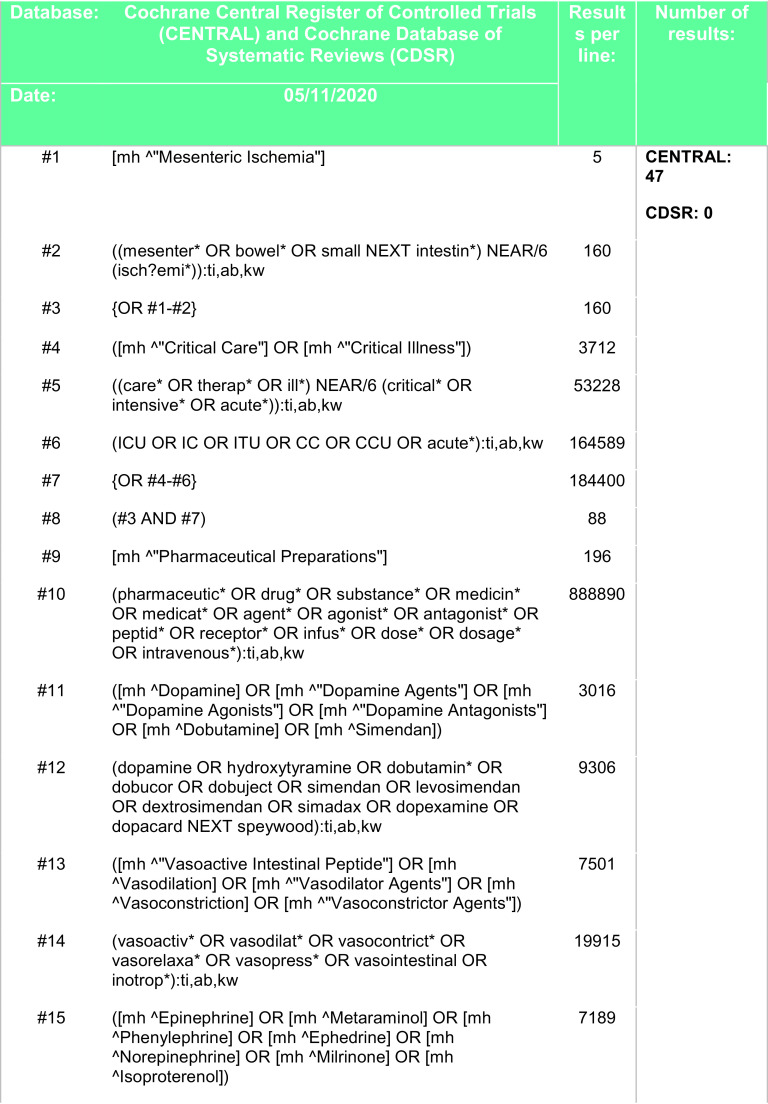

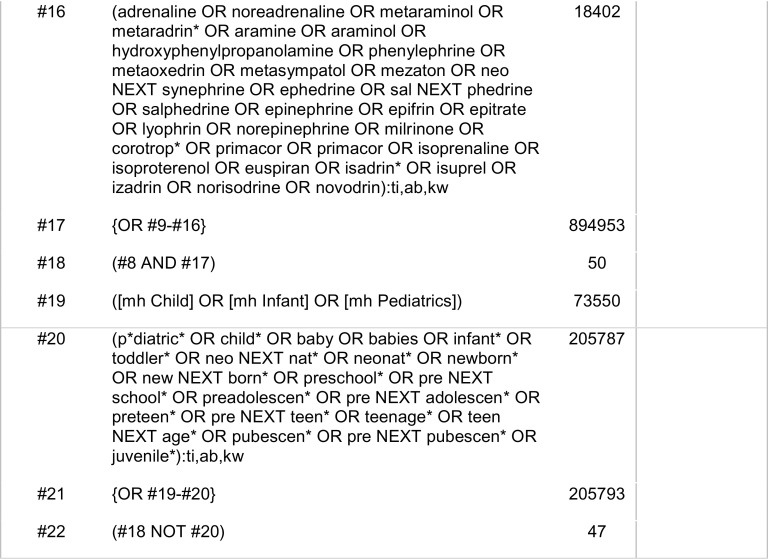
Search strategy - Cochrane Central Register of Controlled Trials (CENTRAL), Cochrane Database of Systematic Reviews (CDSR).

The literature search was conducted by the library department of the Royal College of Surgeons of England. The Patient, Intervention, Control and Outcome (PICO) framework was used and is outlined in Figure 1. Electronic databases of Ovid Medline, EMBASE, Cochrane Central Register of Controlled Trials (CENTRAL) and Cochrane Database of Systematic Reviews (CDSR) were searched for relevant studies. The search was completed on the 5 ^th^ of November 2020 and included all relevant studies since 1946 including non-English studies and case reports. Search strategies included certain drugs, such as noradrenaline, adrenaline, dopexamine, dobutamine, dopamine, levosimendan, vasopressin, ephedrine or phenylephrine were specified. However, freedom of the searchers to add more terms if required was allowed. A combination of keywords and controlled vocabulary was adapted for each database. Search strategies are outlined in Figures 2, 3 and 4.

**Selection process**

Studies were screened for relevant phrases of inclusion, where papers were identified as randomised control trials, performed on non-animal subjects, on the subject relating to the usage of vasoactive drugs in AMI. Papers were screened by four reviewers (CB, PO, EM, PF) independently. The process was facilitated using the Rayyan Platform, allowing for independent and anonymous review and analysis of searched literature. Any discrepancies underwent further review until a consensus was reached.

### Data collection process

Data was collected from the reports identified by the literature search using the Rayyan platform. This platform facilitated independent and anonymous collection of data. 

**Data items**

The Data items we sought were the following: the country of study, the study design, the intervention type (vasoactive medication), pathology and surgery (if any) subtype, the study size, the follow-up-time, the remaining length of small bowel, time to anastomosis, length of critical care stay, length of in hospital stay.

### Risk of bias in individual studies

The reviewers were to assess selected studies using the Grading of Recommendations Assessment, Development and Evaluation (short GRADE) system. GRADE assesses study limitations/risk of bias, inconsistency, indirectness, impreciseness and publication bias. These criteria would have been applied on a study-by-study basis and an outcome level by two reviewers and where there was inconsistency, a third reviewer would assess and provide an outcome. We planned to perform a meta-analysis if the reported results permitted.

### Data synthesis

In the case of a negative search without any eligible randomised controlled trials, the plan was to discuss the existing evidence regarding the problem (AMI) and how the intervention might work (use of vasoactive drugs).

### Meta-bias(es)

The methods were to be reviewed externally to identify possible sources of potential bias.

### Confidence in cumulative evidence

Strength was to be assessed using GRADE tools.

## Results

This systematic review aimed to identify randomised control trials comparing mortality rates in relation to the use of different vasoactive drugs in AMI. Ovid Medline, EMBASE, Cochrane CENTRAL and the Cochrane Database of Systematic Review were systematically searched as outlined in Figures 1- 4.

**Figure 5.**
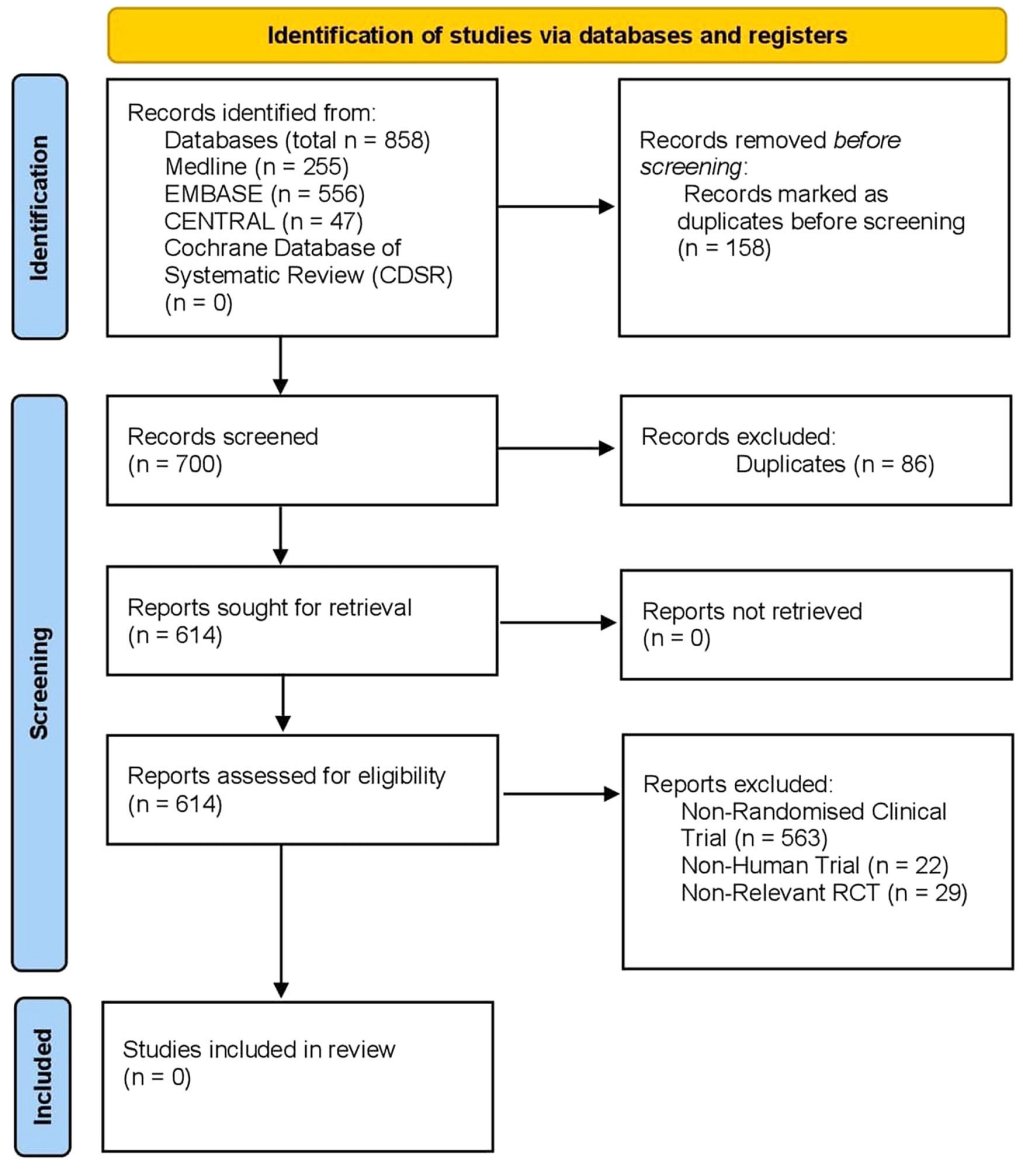
PRISMA flowchart.

The initial search identified a total of 700 articles. After screening for duplicates, 614 articles remained and were reviewed for eligibility. There were no eligible studies identified addressing the specific study question. Reasons for exclusion included: non-randomised controlled trials and studies, non-human trials, and studies which bore no semblance to the question of interest. In view of this, no quantitative analysis was performed. The PRISMA flowchart is outlined in Figure 5.

## Discussion

There is significant variation in the management of AMI, and this may reflect regional variations in mortality associated with this condition. The recent ACPGBI guidance ^24^ on Emergency General Surgery highlighted the relative paucity of research in this field. However, a synthesis of specific aspects of AMI management, such as the choice of vasoactive medications and its influence on mortality was beyond its remit.

This systemic review aimed to ask that question and assessed the previous publications available. It is of interest that this work failed to identify any studies comparing the use of these drugs in AMI against outcomes.

In patients who present with symptoms, clinical findings or imaging suggestive of mesenteric arterial ischaemia, resuscitative measures including the avoidance of systemic hypoxia and intravenous fluids are crucial first steps to optimising blood pressure and end organ perfusion. ^6^ Broad spectrum intravenous antibiotics should also be administered promptly due to the potential for bacterial complications in view of the breach of the mucosal barrier. ^6^ Of the small cohort of patients deemed suitable, urgent revascularisation should be pursued to re-perfuse the ischaemic gut through liaison with the interventional radiologists and vascular surgeons. Most patients who are admitted to critical care units will require vasoactive drugs to optimise their blood pressure and cardiovascular status. However, the ideal drug which gives an appropriate increase in systemic arterial pressure without causing a decrease in or compromise of splanchnic perfusion remains to be elicited and the literature on this question is absent.

### Catecholamines

**Noradrenaline and adrenaline**

In the context of AMI, splanchnic blood flow would seem a salient factor. Pharmacologically, noradrenaline is an endogenous catecholamine which primary has a direct alpha-1 effect, although there is a small degree of β-1 adrenergic agonism. It increases systemic vascular resistance (SVR), increasing afterload;- and causes venoconstriction increasing preload. It is weakly a positive inotrope through its β-1 effect. Some studies suggest that noradrenaline reduces hepato-splanchnic blood flow in septic and non-septic patients. ^25^^,^^26^ Similarly, adrenaline, a sympathomimetic, was found to have a reductive effect on splanchnic blood flow. ^27^

**Dopamine and dopexamine**

Dopamine is a catecholamine, a precursor to noradrenaline, and mediates inotropy via dopamine receptors and vasoconstriction via the alpha-adrenergic pathway. It shows a dose dependent change in action; causing splanchnic dilatation at low doses while increasing SVR at higher doses. Meier-Hellmann *et al.* ^28^ reported an increase in hepato-splanchnic blood flow in septic patients given dopamine although Neviere and colleagues ^29^ reported a decrease in gut mucosal perfusion. Maynard *et al.* ^30^ suggested that dopexamine, a dopamine analogue which has vasodilatory effects, may improve gut microcirculation in septic shock; although subsequent investigators did not confirm these beneficial effects.

**Dobutamine**

Dobutamine, a synthetic catecholamine is a β1-selective adrenoceptor agonist which is utilised clinically as a positive inotrope in the treatment of acute heart failure and cardiogenic shock. Creteur and colleagues ^31^ determined that a dobutamine infusion did improve both splanchnic oxygenation in septic animals and in septic patients. However, Bomberg *et al.* ^32^ suggest that in pigs, dobutamine may improve arteriovenous shunting, but conversely may reduce jejunal mucosal perfusion.

### Non-catecholamines

**Vasopressin**

One observational cohort study found that vasopressin, a potent non-catecholamine vasoconstrictor which acts on vasopressin receptors, improved small bowel perfusion and mortality in patients with non-occlusive mesenteric ischaemia (NOMI) who had undergone cardiopulmonary bypass for elective cardiac surgery. ^13^ However, no further assessment of outcome in AMI appears to have been assessed.

**Levosimendan and milrinone**

Levosimendan is an inotrope which improves contractility by sensitizing cardiac muscle to calcium. It also produces vasodilation by opening ATP-sensitive K+ channels in vascular smooth muscle, although this is not yet demonstrated in the splanchnic circulation.

An experimental study on hypoxic, stressed new-born piglets showed milrinone, a phosphodiesterase inhibitor which produces an inotropic effect and vascular dilation, improves mesenteric perfusion. ^33^

There is thus some basic scientific experimental data available on the effects of some vasoactive agents on the splanchnic circulation. However, there are a number of limitations to this, and none have been extrapolated into clinical trials investigating vasoactive medication in AMI, against other agents. In relation to our study question, most of the experimental data focuses on patients in a shocked state as a result of sepsis, or in elective settings such as planned cardiac surgery, rather than in AMI. None of the studies report any clear data in relation to mortality or morbidity, length of hospital or critical care stay associated with any vasoactive agents.

There may be a role for dobutamine, levosimendan, milrinone, dopamine or vasopressin or other vasoactive agents in improving splanchnic perfusion in mesenteric ischaemia, but further, more extensive, patient-based study is required to elucidate these theories and their clinical significance in relation to patient survival and morbidity.

**Limitations**

This study did not identify any qualifying randomised controlled trials in relation to the study question and therefore did not produce a quantitative analysis.

## Conclusions

This systematic review has identified a gap in literature and research relating to the choice of vasoactive agent in AMI. There are therefore actions we would recommend to aid identifying best practice for this condition. The results of this study would suggest that it is important to investigate current practice and clinician preference. The first step to this would therefore be a Delphi Study which is currently underway and can be found via this link: **https://is.gd/vasoactive_agents_AMI**. Following the survey is an optionable Delphi process.

RCTs where comparison of outcomes with different vasoactive agents is analysed could ultimately improve the care of the critically ill patient with mesenteric ischaemia and remains absent from any work relating to AMI.

## Data availability

All data underlying the results are available as part of the article and no additional source data are required.

## Reporting guidelines

Harvard Dataverse. PRISMA checklist and Review Data for: Vasoactive agents in acute mesenteric ischaemia in critical care. A systematic review. DOI: https://doi.org/10.7910/DVN/2GN0BS. ^34^

Data are available under the terms of the Creative Commons Zero “No rights reserved” data waiver (CC0 1.0 Public domain dedication).
