# Supplementary material for: Vasoactive agents in acute mesenteric ischaemia in critical care.  A systematic review
Source: F1000Res. 2021 Sep 6;10:453. Originally published 2021 Jun 8. [Version 2] doi: 10.12688/f1000research.52782.2 (PMC8459625; doi:10.12688/f1000research.52782.2)
Supplement: Peer review history

# Peer review: Reviewer response for version 2

**DOI:** 10.5256/f1000research.58870.r93548

**Published:** 2021-09-22

**Authors:** Norman Galbraith

**Affiliations:** 1 University Department of Surgery, Glasgow Royal Infirmary, Glasgow, UK; 2 University of Glasgow, Glasgow, UK; 3 NHS Scotland, Glasgow, UK

Thank you for having the opportunity to look at this manuscript again, and for the authors incorporating some of the suggested changes. 

 Most systematic reviews will not have multiple main tables in the article for separate search criteria and the article would be more aesthetic and readable to an audience of they were compressed into one table or better supplemental, but I will leave that to the editors discretion. 

 In one response to a comment from a prior review, there was a suggestion of merit of another review looking at non RCT studies. I would suggest that this subject probably doesn't need multiple separate reviews and that component would be best placed here. However, the aim is clear not to include non RCT studies and this beyond the scope of this review. 

 This article achieves the aim of highlighting a gap in the literature and paves the way for future research in an interesting and understudied subject.

Are the rationale for, and objectives of, the Systematic Review clearly stated?

Yes

Is the statistical analysis and its interpretation appropriate?

Yes

Are sufficient details of the methods and analysis provided to allow replication by others?

Yes

Are the conclusions drawn adequately supported by the results presented in the review?

Yes

Reviewer Expertise:

General surgery, colorectal surgery, Inflammatory response, macrophage/monocyte function

I confirm that I have read this submission and believe that I have an appropriate level of expertise to confirm that it is of an acceptable scientific standard.

# Peer review: Reviewer response for version 1

**DOI:** 10.5256/f1000research.56103.r87010

**Published:** 2021-06-29

**Authors:** Xavier Wittebole

**Affiliations:** 1 Department of Critical Care Medicine, Clinique Universitaire St Luc, Université Catholique de Louvain, Brussels, Belgium

**GENERAL COMMENTS:**

 The authors performed a systematic review to assess the role of vasopressors in acute mesenteric ischemia (AMI). From 614 papers, they could not find any randomized controlled trial and conclude “ *Multicenter randomized controlled trials comparing these medications on clinical outcomes will therefore be required to address this question*.” They further discuss the effects of some of these vasoactive agents.

**SPECIFIC COMMENTS:**

- The authors distinguish 2 different mechanisms that may explain AMI and propose vasopressors would be responsible for non-occlusive MI (NOMI). However, in NOMI, it is difficult to assess the exact role of vasopressors versus the cause for which the vasopressors are used. For instance, the microcirculatory changes observed in sepsis may also explain some ischemic lesions without any effect of vasopressors per se. This could be briefly discussed.
- The authors should also better clarify the fact they searched for the best vasopressor therapy in AMI versus the medication that would be responsible for the development of AMI.
- A recent meta-analysis (Belletti *et al. *2020 ^1^) assessed the effects of continuous epinephrine infusion on survival in critical care patients. In this MA, a secondary endpoint evaluated the development of bowel ischemia. This study might be a source of other papers that could be assessed for this present study.
- It might be of interest the authors check some large trials on vasopressors (vasopressin and the Vasst study; selepressin and the SepsisAct study; angiotensin II and the Athos-3 study; etc). By evaluating the side effects in those trials (mesenteric ischemia), the authors could already provide a beginning of answer to the question raised. While out of the scope of this study, this might however be of interest.
- When discussing the various vasoactive drugs, I would propose the authors classify them as “mostly vasopressors” (nor-adrenalin, etc) and “mostly inotropes” dobutamine, etc) and “mixed action” (epinephrine, etc).
- Some vasopressors are not discussed. (terlipressin, selepressin, angiotensin II).

 

**MINOR COMMENTS:**

- I personally found the abstract’s background too long.
- Figures 2, 3 and 4 could be provided in a supplementary appendix.
- The authors could also remind the reader that CT scan has a low sensitivity to diagnose trans-mural ischemia (see for instance Verdot *et al. *(2021 ^2^)).
- In the Eligibility Criteria paragraph, the authors propose various secondary outcomes including “length of critical care admission”. I would propose to write “ICU length of stay”.

Are the rationale for, and objectives of, the Systematic Review clearly stated?

Yes

Is the statistical analysis and its interpretation appropriate?

Yes

Are sufficient details of the methods and analysis provided to allow replication by others?

Yes

Are the conclusions drawn adequately supported by the results presented in the review?

Yes

Reviewer Expertise:

Critical Care Medicine, Sepsis

I confirm that I have read this submission and believe that I have an appropriate level of expertise to confirm that it is of an acceptable scientific standard, however I have significant reservations, as outlined above.

## References

[1] : Effect of Continuous Epinephrine Infusion on Survival in Critically Ill Patients: A Meta-Analysis of Randomized Trials. *Crit Care Med* .48(3) : 10.1097/CCM.0000000000004127 398-405 10.1097/CCM.0000000000004127 31789701

[2] : Diagnostic performance of CT for the detection of transmural bowel necrosis in non-occlusive mesenteric ischemia. *Eur Radiol* .2021; 10.1007/s00330-021-07728-w 10.1007/s00330-021-07728-w 33585993

# Peer review: Reviewer response for version 1

**DOI:** 10.5256/f1000research.56103.r87013

**Published:** 2021-06-21

**Authors:** Norman Galbraith

**Affiliations:** 1 University Department of Surgery, Glasgow Royal Infirmary, Glasgow, UK; 2 University of Glasgow, Glasgow, UK; 3 NHS Scotland, Glasgow, UK

This is a systematic review of which vasoactive therapeutics are best in patients with acute mesenteric ischaemia. This is a clinically important and under-researched condition with a poor evidence base. I applaud the authors for raising pursuing this topic and this article is an important step to highlight the lack of evidence, despite being so common, in a systematic way. The article is well written and understandable. Methodologically, it follows the PRISMA steps of a systematic review.

 Some suggestions I have are:

1. It wasn’t entirely clear to me whether this study was to determine which agents were optimum in part of the treatment of acute mesenteric ischaemia (i.e. occlusive), or which agent was a potential cause of acute mesenteric ischaemia in the already critically unwell patient with another primary diagnosis e.g. sepsis. I would suggest clarifying this when stating the aim/hypothesis at the end of the introduction, or in the methods section (and potentially in the “Research question” section of Figure 1 by including the phrase “the treatment of” or “prevention of”). 
2. As a clinically important, common but well known to be under-researched area, it is maybe not surprising there are no RCT’s in this subject. Did the authors consider widening their search to include non-randomised prospective studies, or observational/retrospective studies where we might expect some evidence to lie? If not, due to the likely biased nature of some of these weaker type of studies, I would suggest the authors state/justify why they have not included this body of evidence in their study. Alternatively, if the authors have searched this or have some data of observational studies, adding this as a table or supplementary table would strengthen the paper.
3. Having the exact search strategy included is appreciated and makes the study more reproducible, however, I would suggest putting all of these tables to the supplementary section.
4. To further the strength of the methodology, demonstrating that the “grey literature” has been searched to check for additional evidence/publication bias would be helpful. Some articles now use the OpenGrey platform to do this.

Are the rationale for, and objectives of, the Systematic Review clearly stated?

Yes

Is the statistical analysis and its interpretation appropriate?

Yes

Are sufficient details of the methods and analysis provided to allow replication by others?

Yes

Are the conclusions drawn adequately supported by the results presented in the review?

Yes

Reviewer Expertise:

General surgery, colorectal surgery, Inflammatory response, macrophage/monocyte function

I confirm that I have read this submission and believe that I have an appropriate level of expertise to confirm that it is of an acceptable scientific standard, however I have significant reservations, as outlined above.

# Response

**Published:** 2021-08-24

**Authors:** Christopher Brennan

**Affiliations:** Aberdeen Royal Infirmary, UK

- *It wasn’t entirely clear to me whether this study was to determine which agents were optimum in part of the treatment of acute mesenteric ischaemia (i.e. occlusive), or which agent was a potential cause of acute mesenteric ischaemia in the already critically unwell patient with another primary diagnosis e.g. sepsis. I would suggest clarifying this when stating the aim/hypothesis at the end of the introduction, or in the methods section (and potentially in the “Research question” section of Figure 1 by including the phrase “the treatment of” or “prevention of”). *
- **Response: **Our review intends to look at vasoactive choice in treatment of mesenteric ischaemia, as opposed to risk of causing ischaemia. Although ischaemia as side effect of vasoactive agents was not considered here, it is of interest to consider both for the pathophysiology and for the evaluation of the most promising agent for a future RCT. We have clarified this in the aims section of the introduction.
- *As a clinically important, common but well known to be under-researched area, it is maybe not surprising there are no RCT’s in this subject. Did the authors consider widening their search to include non-randomised prospective studies, or observational/retrospective studies where we might expect some evidence to lie? If not, due to the likely biased nature of some of these weaker type of studies, I would suggest the authors state/justify why they have not included this body of evidence in their study. Alternatively, if the authors have searched this or have some data of observational studies, adding this as a table or supplementary table would strengthen the paper.*
- **Response: **Many thanks for your comment. For the purpose of this review, only RCT’s were considered. A further review in the future with our search protocol, analysing case reports, would likely be of merit. Our review highlights the lack of RCT’s on the subject matter, which can be used to prompt further analysis of data. We have added a statement to the conclusion.
- *Having the exact search strategy included is appreciated and makes the study more reproducible, however, I would suggest putting all of these tables to the supplementary section.*
- **Response:** Many thanks. The structure of this systematic review is as per the publishers guidelines.
- *To further the strength of the methodology, demonstrating that the “grey literature” has been searched to check for additional evidence/publication bias would be helpful. Some articles now use the OpenGrey platform to do this.*
- **Response:** Many thanks for your comment. However, we have not performed a grey literature search as we wish to assess if there is any direct comparison published, in the form of RCT between different vaso-active agents in this setting.

# Peer review: Reviewer response for version 1

**DOI:** 10.5256/f1000research.56103.r87014

**Published:** 2021-06-14

**Authors:** Nick Heywood

**Affiliations:** 1 East Lancashire Hospitals NHS Trust, East Lancashire, UK

I would like to thank the authors for the opportunity to review this paper “Review of Vasoactive agents in acute mesenteric ischaemia in critical care.  A systematic review." This is indeed an area where there is paucity of research and this paper certainly demonstrates that. I do however have the following points which should be addressed before acceptance for indexing in Pub Med.

- The authors state that their aim in the introduction is: “ *This primary aim of this systematic review is to evaluate the current evidence comparing mortality outcomes for vasoactive drugs in AMI”, *however in the results section they state that *“This systematic review aimed to identify randomised control trials comparing mortality rates in relation to the use of different vasoactive drugs in AMI.”* The authors state in the abstract results section that there is no randomised controlled trial, and this is the only result. The authors included non-randomised trials including case reports in their search, yet there is no comment on the results, nor any narrative as to the types of study out of the 614 returned in the search. There should be a descriptive in the result section as to the numbers of each types of study, i.e. number of case reports (including number of patients in these case reports), number of observational studies etc.
- The Methods section is very detailed and could be easily replicated. I commend the authors for their thorough search of the all the relevant articles.
- With regards to the findings, they do indeed make this study difficult to write in a manuscript and the authors need to be clear about searching for rCTs vs case inclusion of case reports. For example, if the authors were not interested in case reports, why were they not excluded early in the study. It may be that they wanted to ensure no studies were missed, but this should be discussed in the paper.
- The findings, or lack thereof, makes the discussion limited. Upon reading the discussion, it felt like reading a review article rather than the discussion section of a systematic review. I understand that the authors need to write the discussion, but much of this does not seem relevant to the discussion section of this type of article. It feels more at place in an introduction or a separate article regarding the choices of treatments for this disease process.
- The authors should focus on discussing the types of papers that were found in the review, what types of studies they found and their limitations. The should also discuss why some of the 29 non-relevant RCTs were indeed non-relevant. Although the authors touch upon this in the introduction, they may wish to focus the discussion on the difficulties associated with undertaking this type of study and why there may be lack of data. A degree of speculation is needed here, but would be more relevant than the pharmacology of vasoactive drugs, for which should not really be included in the discussion section.

Overall, I agree that this message is very important and certainly supports future work, that I am glad to see is being developed, however, this paper needs more work before acceptance for indexing in PubMed

Are the rationale for, and objectives of, the Systematic Review clearly stated?

Partly

Is the statistical analysis and its interpretation appropriate?

Not applicable

Are sufficient details of the methods and analysis provided to allow replication by others?

Yes

Are the conclusions drawn adequately supported by the results presented in the review?

Yes

Reviewer Expertise:

My areas of expertise include general surgery with specific interest in collaborative research, colorectal sruegry and in particular pelvic floor disorders and cancer

I confirm that I have read this submission and believe that I have an appropriate level of expertise to confirm that it is of an acceptable scientific standard, however I have significant reservations, as outlined above.

# Response

**Published:** 2021-08-24

**Authors:** Christopher Brennan

**Affiliations:** Aberdeen Royal Infirmary, UK

- *The authors state that their aim in the introduction is: “This primary aim of this systematic review is to evaluate the current evidence comparing mortality outcomes for vasoactive drugs in AMI”, however in the results section they state that “This systematic review aimed to identify randomised control trials comparing mortality rates in relation to the use of different vasoactive drugs in AMI.” The authors state in the abstract results section that there is no randomised controlled trial, and this is the only result. The authors included non-randomised trials including case reports in their search, yet there is no comment on the results, nor any narrative as to the types of study out of the 614 returned in the search. There should be a descriptive in the result section as to the numbers of each types of study, i.e. number of case reports (including number of patients in these case reports), number of observational studies etc. Our primary interest in undertaking this work was to assess whether there was capacity for a randomised clinical trial to be developed comparing different vasoactive agents in this field. Thus, for this systematic review only RCT’s were considered for inclusion.*
- **Response: **The search protocol identified many non-RCT’s, which were excluded at an early stage and not further analysed. The further RCT’s were excluded for being entirely unrelated, or trials not performed in humans. We have edited the results section to clarify this. We have also added a descriptor in the results section highlighting the breakdown of results, and reasons for exclusion.
- *The Methods section is very detailed and could be easily replicated. I commend the authors for their thorough search of the all the relevant articles.*
- **Response: **We thank the reviewer for their commendation.
- *With regards to the findings, they do indeed make this study difficult to write in a manuscript and the authors need to be clear about searching for rCTs vs case inclusion of case reports. For example, if the authors were not interested in case reports, why were they not excluded early in the study. It may be that they wanted to ensure no studies were missed, but this should be discussed in the paper. Case reports were identified in the initial search protocol and thus acknowledged. However as we were specifically interested in RCT’s, these were excluded.*
- **Response:** As the reviewer suggests, we did not want to miss any studies and this is why we did not remove case reports initially. This fact has been further highlighted in the methods section.
- *The findings, or lack thereof, makes the discussion limited. Upon reading the discussion, it felt like reading a review article rather than the discussion section of a systematic review. I understand that the authors need to write the discussion, but much of this does not seem relevant to the discussion section of this type of article. It feels more at place in an introduction or a separate article regarding the choices of treatments for this disease process.*
- **Response:** Given the results of our systematic review we felt that discussion around the broader topic would be of benefit to provide context to the relevance of our review. Due to the review only specifically looking for RCT’s, further analysis of the case reports identified was not performed. This is an area of research that, with our search strategy freely available, could be performed in the future. We have added this statement to the conclusion section.
- *The authors should focus on discussing the types of papers that were found in the review, what types of studies they found and their limitations. The should also discuss why some of the 29 non-relevant RCTs were indeed non-relevant. Although the authors touch upon this in the introduction, they may wish to focus the discussion on the difficulties associated with undertaking this type of study and why there may be lack of data. A degree of speculation is needed here, but would be more relevant than the pharmacology of vasoactive drugs, for which should not really be included in the discussion section.*
- **Response: **Of the 29 human non-relevant RCT’s, none were on the subject of mesenteric ischaemia, or vasoactive support. We have specifically clarified this point in the discussion section.
